# Surgical Treatment of a Supratentorial Extraventricular Ependymoma: A Case Report

**DOI:** 10.7759/cureus.40196

**Published:** 2023-06-09

**Authors:** Jesús E Falcón, Joel A Velázquez, Ricardo García, Iván Téllez, Marco A Rodríguez

**Affiliations:** 1 Department of Neurosurgery, Hospital de Especialidades, Centro Médico Nacional Siglo XXI, Instituto Mexicano del Seguro Social, Mexico City, MEX; 2 Department of Pathology, Hospital de Especialidades, Centro Médico Nacional Siglo XXI, Instituto Mexicano del Seguro Social, Mexico City, MEX

**Keywords:** glial tumor, gross total resection, ependymoma, extraventricular, supratentorial

## Abstract

Supratentorial extraventricular ependymomas (STEE) are very rare primary tumors of the central nervous system (CNS). A 19-year-old man complained of headache, hemiparesis and seizures and was admitted to our hospital. Magnetic resonance imaging (MRI) revealed a right frontal intra-axial lesion. The patient underwent surgical treatment, and the tumor was resected successfully. A diagnosis of World Health Organization (WHO) grade 3 STEE was based on microscopic examination and immunohistochemical analysis. The patient was discharged without a neurological deficit.

## Introduction

Ependymomas are relatively uncommon glial tumors that account for 3% of primary central nervous system (CNS) tumors in adults [1]. They can involve any of three compartments of the CNS: supratentorial, posterior fossa, and spinal cord, and affect all age groups [2]. Although most ependymomas are localized in the posterior fossa, only 30% are supratentorial. Extraventricular ependymomas represent 39% of the supratentorial compartment [3]. These ependymoma variants are labeled extraventricular, ectopic, cortical, or lobar [4].

## Case presentation

A 19-year-old male with no significant past medical history was admitted to the emergency department. He presented two events of 10 minutes duration of focal motor fits involving the right superior limb which became generalized with tonic-clonic seizures and somnolence in postictal status. He complained of episodes of frontal, oppressive and moderate-intensity headaches associated with nausea and vomiting over one month before admission. In the previous two weeks, the patient was referred to progressive left-sided weakness. His neurological examination revealed mild left-sided paresis without any other neurological deficit. A complete blood count, liver function test, basic blood chemistry test, and coagulation test were performed. The results of the serum analysis were normal.

**Figure 1:**
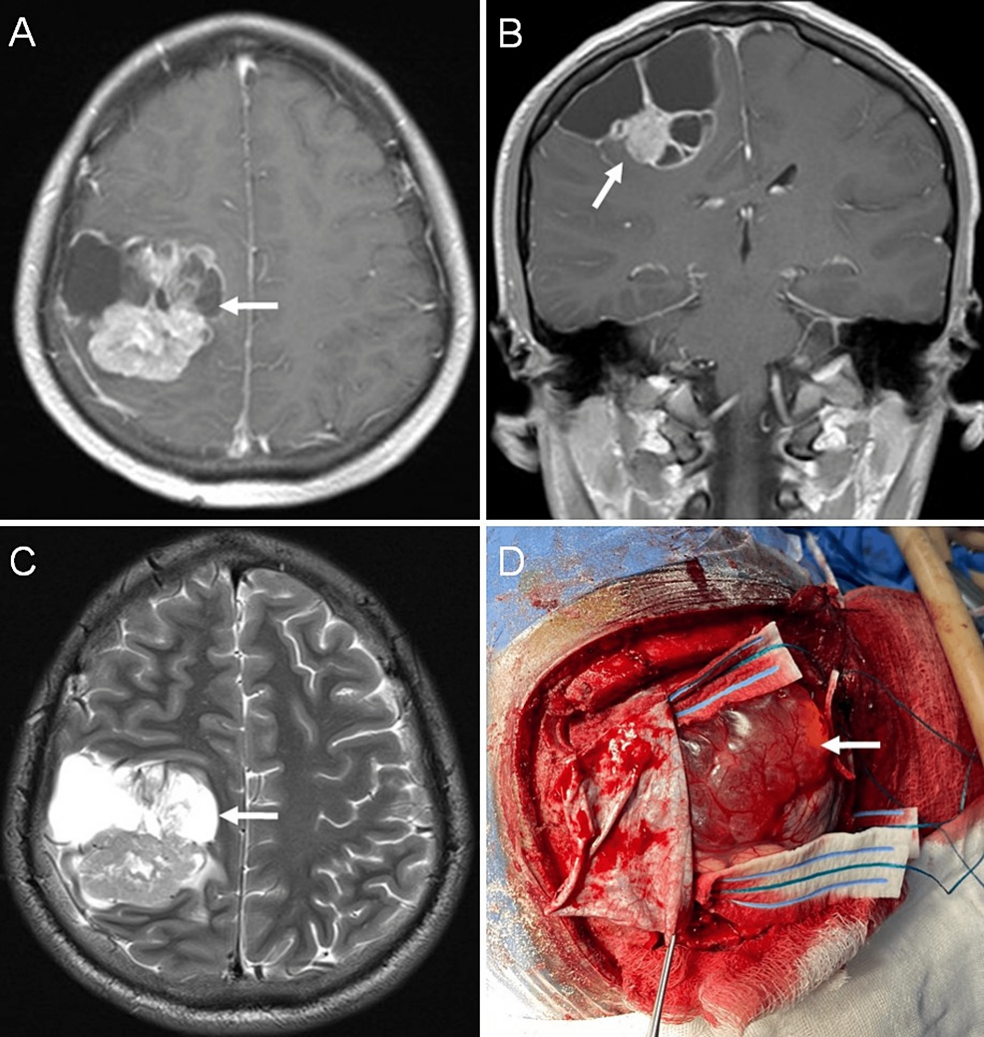
Preoperative MRI images and intraoperative macroscopic surgical view (A, B) Axial T1-weighted MRI section with contrast demonstrating a supratentorial extraventricular lesion (white arrow). (C) Axial T2-weighted section shows solid-cyst component (white arrow). (D) Surgical view shows a cystic cortical lesion after durotomy (white arrow).

A cranial magnetic resonance imaging (MRI) showed a right fronto-parietal intra-axial and well-defined multilobulated lesion, measuring 42 x 47 x 55 mm with perilesional edema. The lesion had a cyst component with hyposignal on T1 and hypersignal on T2-weighted images and showed restricted diffusion. After gadolinium administration, it showed heterogeneous enhancement with intensely periferic enhancing of the cystic portion (Figures 1A-1C).

Under general anesthesia in supine position with the head fixed with the Mayfield skull clamp and 45° grades rotated to left, a horse shoe incision and a right parasagittal craniotomy bone flap was created. The dura was opened in square-shaped and was based on the superior sagittal sinus. The tumor was found to be a solid-cystic lesion. The cystic portion was surfacing at a large parasagittal area and the deeper walls were found to be surrounded by gliotic brain tissue. An adequate line of cleavage was found and we proceed to dissection under surgical microscope. Gross total resection was achieved and hemostasis was secured (Figure 1D).

**Figure 2:**
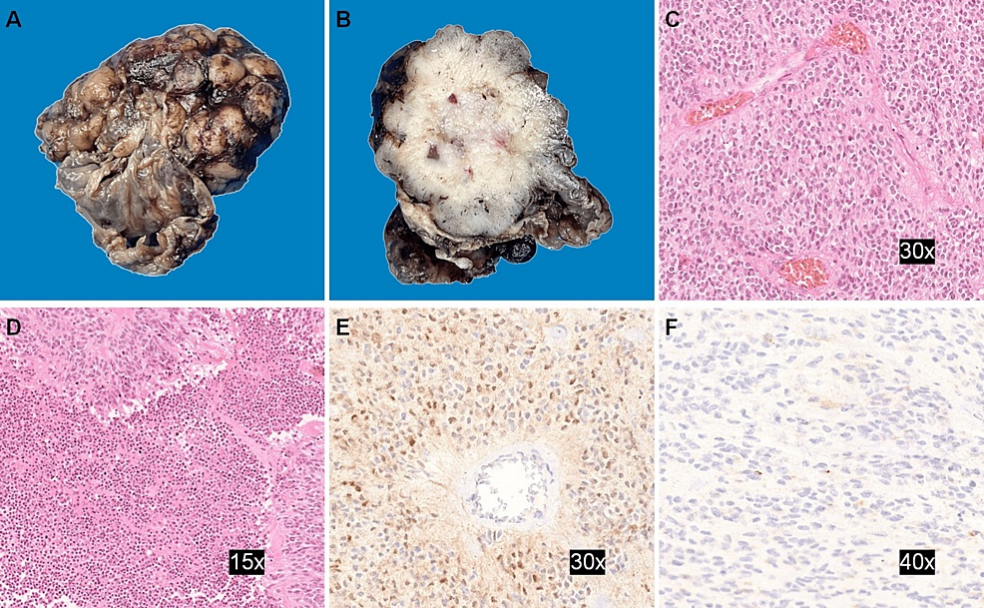
Pathological analysis (A, B) The macroscopic view reveals a solid-cystic tumor with a cerebriform surface. (C, D) The hematoxylin and eosin stain shows an ependymal neoplasm with the formation of pseudorosettes and necrotic zone. (E) Nuclear cyclin D1 expression. (F) Expression of epithelial membrane antigen.

Pathological analysis was performed, and macroscopic examination revealed a solid-cyst aspect, brownish and cerebriform shape neoplasm (Figures 2A, 2B). Histological findings showed on hematoxylin and eosin stain a glial neoplastic lesion with ependymal differentiation, abundant pseudorosettes, hypercellularity with necrosis foci and hyperplasia endothelial. The cells were median, oval-oligoid shape, with scarce cytoplasm and round nucleus with “salt and pepper” chromatin distribution. It presented mitotic activity of four mitoses per 10 fields (objective x 40) and cellular proliferation index (Ki 67) of 15%. Many cells exhibit expression of nuclear cyclin D1 and epithelial membrane antigen (Figures 2C-2F).

**Figure 3:**
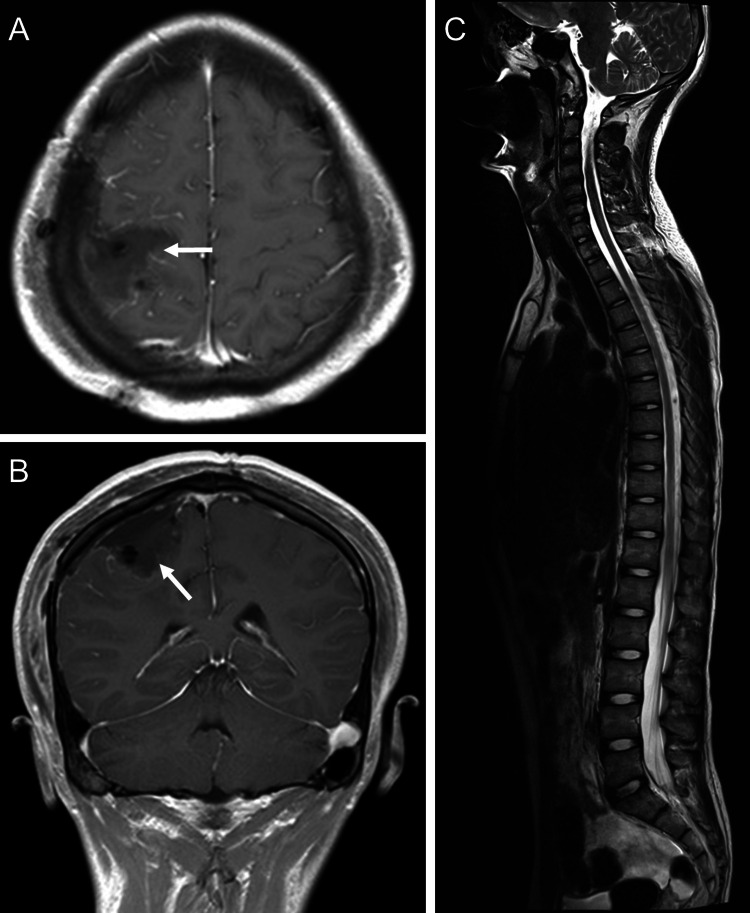
Postoperative MRI images (A, B) Post-surgical MRI in an axial T1-weighted section with contrast shows total tumor resection (white arrows). (C) Craniospinal MRI in a sagittal T2-weighted section without evidence of metastatic lesions.

The patient´s hemiparesis improved, and he was discharged in a stable condition after four days of hospital stay. At two months follow-up, he had resolution of all symptoms. On postoperative cranioespinal MRI there was no evidence of residual tumor or other lesions at spinal level (Figures 3A-3C). He was admitted to radiotherapy service to receive adjuvant radiotherapy (RT) at our center.

## Discussion

Ependymomas represent 3%-4% of all intracranial neuroepithelial neoplasms of CNS in adults [1,5]. The current WHO classification of CNS tumors (2021) classified ependymomas according to a combination of histopathological and molecular features as well as anatomic sites. This classification lists two molecularly defined types of supratentorial ependymoma: zinc finger translocation-associated (ZFTA) fusion and YAP1 fusion (yes-associated protein 1) [6]. The term “anaplastic” ependymoma is no longer indexed [7].

In adults, intracranial ependymomas represent 19% and have a predilection for supratentorial level and the majority are classified as WHO Grade 3 [8]. The molecular study was not possible to perform in our hospital. However, the case we presented is about a WHO grade 3 ependymoma that expresses Cyclin D1. ZFTA gene rearrangements induce the hyperexpression of L1 cell adhesion molecule (L1CAM) and/or cyclin D1 expression may be considered as a surrogate marker of the ZFTA group [9].

The pathogenesis remains unclear, it has been proposed supratentorial extraventricular ependymoma (STEE) develops from the rest of the ependymal cells. Vernet et al. proposed that the origin of STEE could be developed from 1) an ependymal cyst; 2) an outpouched ependymal cell lining; and 3) a primitive neuroectodermal tumor which would differentiate along the ependymal lineage [10].

The STEE are frequently localized in frontal and temporal lobes. Clinical manifestations are unspecific and depend on the localization and tumor size. The common presenting signs and symptoms of intracranial ependymomas are headache, seizure, and motor weakness [11].

The STEE usually show a heterogeneous appearance on T1-T2 weighted sequences and tends to be large lesions at presentation, especially of the high-grade variety. The majority of lesions show a solid cystic appearance that tends to compress rather than infiltrate the surrounding brain parenchyma. An important characteristic that differentiates other gliomas and STEE is a well-defined demarcation line between tumor and peripheral brain tissue, and it can be seen both radiologically and intraoperatively [12].

STEE are tumors that generate controversy in their clinical management. In literature, reported factors of poor prognosis of STEE include age, histology, tumor size, extent of resection and adjuvant RT. Wang et al. identified EOR and tumor grade are both prognostic factors for overall survival (OS) and progression-free survival rates (PFS). Subtotal resection and WHO grade 3 ependymomas predicted worst OS and PFS [13]. Goya et al. noted that the presence of the ZFTA fusion is not associated with prognostic value in terms of OS or PFS. Only gross total resection correlates with better outcomes [14].

The aim in the management of STEE is gross total resection and in cases of recurrence, reoperation should be considered. The local recurrence is the most common recurrence pattern. The role of adjuvant RT in treatment of STEE is controversial. It is widely accepted that postoperative RT should be administered especially in patients with WHO grade 3 and those with subtotal resection [13,15].

Ependymomas can spread through cerebrospinal fluid in 11.2% with STEE. Many authors consider all patients to undergo craniospinal screening with a magnetic resonance imaging study [16].

## Conclusions

STEE are infrequent glial tumors. The WHO grade 3 ependymomas tend to have a local recurrence and are worst prognostic compared with infratentorial or spinal lesions. The extension of resection and tumor grade are the major factors for prognosis. Gross total resection extension and adjuvant RT were the modalities of treatment in this case. A craniospinal MRI must be performed to exclude other lesions. Close follow-up is required to monitor the recurrence or progression of the disease.

## References

[1] Armstrong TS, Vera-Bolanos E, Bekele BN, Aldape K, Gilbert MR (2010). Adult ependymal tumors: prognosis and the M. D. Anderson Cancer Center experience. Neuro Oncol.

[2] Wu J, Armstrong TS, Gilbert MR (2016). Biology and management of ependymomas. Neuro Oncol.

[3] Schwartz TH, Kim S, Glick RS (1999). Supratentorial ependymomas in adult patients. Neurosurgery.

[4] Kalfas F, Scudieri C (2019). World Health Organization Grade III supratentorial extraventricular ependymomas in adults: case series and review of treatment modalities. Asian J Neurosurg.

[5] Villano JL, Parker CK, Dolecek TA (2013). Descriptive epidemiology of ependymal tumours in the United States. Br J Cancer.

[6] Louis DN, Perry A, Wesseling P (2021). The 2021 WHO classification of tumors of the central nervous system: a summary. Neuro Oncol.

[7] Ellison DW, Aldape KD, Capper D (2020). cIMPACT-NOW update 7: advancing the molecular classification of ependymal tumors. Brain Pathol.

[8] Hübner JM, Kool M, Pfister SM, Pajtler KW (2018). Epidemiology, molecular classification and WHO grading of ependymoma. J Neurosurg Sci.

[9] Zaytseva M, Papusha L, Novichkova G, Druy A (2021). Molecular stratification of childhood ependymomas as a basis for Personalized Diagnostics and treatment. Cancers (Basel).

[10] Vernet O, Farmer JP, Meagher-Villemure K, Montes JL (1995). Supratentorial ectopic ependymoma. Can J Neurol Sci.

[11] Sun S, Wang J, Zhu M (2018). Clinical, radiological, and histological features and treatment outcomes of supratentorial extraventricular ependymoma: 14 cases from a single center. J Neurosurg.

[12] Jabeen S, Konar SK, Prasad C (2020). Conventional and advanced magnetic resonance imaging features of supratentorial extraventricular ependymomas. J Comput Assist Tomogr.

[13] Wang M, Zhang R, Liu X (2018). Supratentorial extraventricular ependymomas: a retrospective study focused on long-term outcomes and prognostic factors. Clin Neurol Neurosurg.

[14] Goyal-Honavar A, Balasundaram A, Thayakaran IP (2022). ZFTA (zinc finger translocation associated) fusion in supratentorial ependymomas: low prevalence in South Asians and no correlation with survival. World Neurosurg.

[15] Byun J, Kim JH, Kim YH, Cho YH, Hong SH, Kim CJ (2018). Supratentorial extraventricular ependymoma; retrospective analysis of 15 patients at a single institution. World Neurosurg.

[16] Calvo FA, Hornedo J, De La Torre A (1983). Intracranial tumors with risk of dissemination in neuroaxis. Int J Radiat Oncol Biol Phys.

